# Tachometric Cup Anemometer with Wind Direction Indicator and Fibre-Optic Signal Transmission

**DOI:** 10.3390/s25113281

**Published:** 2025-05-23

**Authors:** Paweł Ligęza, Paweł Jamróz, Katarzyna Socha

**Affiliations:** Strata Mechanics Research Institute, Polish Academy of Sciences, Reymonta 27, 30-059 Krakow, Poland; jamroz@imgpan.pl (P.J.); socha@imgpan.pl (K.S.)

**Keywords:** wind velocity and direction, tachometric anemometer, optical–mechanical converter, fibre-optic data transmission

## Abstract

This article presents an innovative design of a tachometric anemometer for measuring wind velocity and direction, which does not contain electronic components and systems or power supply systems in the measurement area. This device can be used in extremely unfavourable environmental operating conditions, in locations exposed to direct atmospheric discharges, in conditions requiring restrictive and intrinsic safety, in special military applications, and in measurements in the presence of extreme electromagnetic fields. An innovative optical–mechanical transducer is used in the anemometer. This transducer generates a light pulse signal, the frequency of which is a function of the flow velocity, and the duty cycle is a function of the wind direction. This signal is transmitted via optical fibre from the sensor assembly to the measuring station, located outside the measurement area. The design of the device is simple, durable, and resistant to environmental conditions.

## 1. Introduction

The atmosphere, which has a mass of only one millionth of the mass of our planet [1], is a key element determining the course of most processes and phenomena important for our life on Earth. An inherent feature of air, i.e., the mixture of gases and aerosols that make up the atmosphere, is constant movement. This movement, which is generally turbulent, can be treated for many utilitarian purposes as somewhat ordered, in which air masses move at a specific speed and direction. In particular, air movements in the horizontal plane, referred to as wind, have long been an object of observation, monitoring, and use by humans. Today, in fields such as meteorology [2,3,4,5] and renewable wind energy [6,7,8,9,10,11], measuring wind velocity and direction is one of the most important metrological issues. However, the area of issues in which knowledge of air flow parameters is important is very extensive. Devices used to measure air velocity, called anemometers, are used in many areas of human activity. These, among others, include land, sea, and air transport, energy, and industrial processes, construction, environmental protection, air conditioning and ventilation, agriculture and breeding, and medicine. In all these and many other areas, measuring air flow velocity is necessary to monitor, control, and optimize the processes taking place there [12].

Anemometers are many types of tools, devices and systems that measure air flow parameters, but use different principles of operation based on various physical phenomena. The phenomena used here, the course of which depends on many physical parameters, include flow velocity. In knowing the model of the phenomenon and measuring the parameters of its course, the measured flow velocity is determined. These phenomena include pressure effects, ultrasound propagation, heat exchange, Karman vortices, the propagation of markers in the flow, and momentum transfer.

In pressure methods, flow velocity is measured by measuring the effect of pressure change on the measuring element placed in the flow. These elements include orifices, nozzles, capillaries, Venturi nozzles, Pitot tubes, and Prandtl tubes [13,14,15,16]. Since physical phenomena are of a basic nature here, these methods can be used as reference methods. Ultrasonic anemometers use the propagation of sound waves in the flow. These devices have a set of ultrasound transmitters and receivers. Measuring the propagation time of the sound wave between the source and the detector allows for determining the flow velocity [17,18,19,20,21,22,23,24,25,26]. In an advanced measurement system with an appropriate space configuration of ultrasound transmitters and receivers, it is possible to measure the 3D velocity vector. Calorimetric anemometers are devices that use the phenomenon of heat transfer between the heated measuring sensor of the anemometer and the flow. The heat flux transferred from the sensor to the flow is a measure of velocity. This flux is measured by measuring the power of the sensor heating electrical current and the temperature of the measuring element. These anemometers require calibration in a reference flow [27,28,29,30,31,32,33,34,35,36,37,38,39,40,41,42]. Placing a suitably selected obstacle in the flow causes the formation of Karman vortices behind the obstacle. This phenomenon is also used for anemometric measurements. The measured flow velocity is related to the frequency of the vortices formed [43,44,45,46]. The flow velocity can also be determined by measuring the velocity of naturally existing or artificially introduced markers into the flow. Markers can be objects transported with the flow at a similar velocity, the motion of which can be measured. We distinguish, among others, mechanical, ionization, chemical, thermal, and optical markers. Laser Doppler Anemometers [47,48,49,50,51,52,53] are instruments for measuring the flow velocity indirectly, by measuring the frequency of light flashes reflected from optical markers moving with the flow through a system of equidistant interference fringes of laser light. Particle Image Velocimetry [54,55,56,57,58,59,60] uses the sequential photography of optical markers in a flow cross-section illuminated by laser light. The correlation function of subsequent images allows for determining the vectors of marker movement. This method allows for determining the flow velocity field in the studied area.

Important classes of devices designed to measure air flow velocity in technical and industrial applications are tachometric anemometers with a rotating measuring element. There are two main types of such devices: vane and propeller anemometers, in which the measuring element rotates in a plane perpendicular to the measured velocity vector, and cup anemometers, in which the measuring element rotates in a plane parallel to the measured flow velocity vector. Tachometric anemometers operate based on the phenomenon of momentum exchange between the tested flow and the measuring rotor. The flow velocity is determined based on the measurement of the rotor’s rotational speed. These anemometers are widely used in flow metrology, and the main application area is meteorological, energy, and ventilation measurements. Tachometric anemometers have many advantageous metrological properties, such as a wide range of measured velocities, characteristics close to linear, low sensitivity to changes in the other physical parameters of the medium, measurement averaging properties, and the durability and reliability of the structure. Tachometric anemometers also have drawbacks. As mechanical systems with moving elements, they are subject to wear over time. In addition, these anemometers are sensitive to environmental factors such as precipitation, pollution, low and high temperatures, and solar radiation. These factors affect the changes in the metrological characteristics of the devices. An important problem in the design of tachometric anemometers is the selection of appropriate bearings and their covers. Bearings should be durable, be protected against external factors, and introduce the smallest possible friction resistance into the measuring system. Friction is a decisive factor influencing the minimum measuring speed and the sensitivity of the anemometer. This type of anemometer is subject to continuous research, optimization, and improvement, and innovative designs are developed for specialized applications.

Article [61] presents the collective results of a long-term research program of cup anemometers. Such anemometers used for industrial measurements require continuous control and standardization. The research involved large series of calibrations and analyses of anemometer parameters, examining the effect of various environmental and operational factors on measurement results. Article [62] is devoted to an innovative concept of optimizing the dynamic properties of an anemometer with a rotating measuring element. This optimization method consists of equipping the device with additional electronic rotor acceleration and braking systems, controlled by the derivative of the instantaneous value of angular velocity. The use of this method allows for a ten-fold reduction in the error in measuring the average velocity in fluctuating flows. The innovative design of a cup anemometer, entirely manufactured using 3D printing technology, is discussed in article [63]. The additive printing of a hexagonal mechanical structure made of ABS material was used here. This technology can be used to produce a small batch of anemometers with a design and dimensions dedicated to a specialized, individual measurement problem. Another innovative design of a tachometric anemometer was described in article [64]. The device is distinguished by the fact that it has a maintenance-free, autonomous power supply. A triboelectric nanogenerator driven by the rotational energy of the measuring rotor is used to power the anemometer. This solution, combined with the wireless transmission of measurement data, enables the multi-point monitoring of the air flow velocity field in large-size objects. Article [65] presents an innovative concept and the results of preliminary tests of an algorithm for correcting dynamic errors of tachometric anemometers. The method uses precise measurement of the rotor rotational speed. The correction algorithm is based on the dynamic model of the anemometer and allows for extending the transfer band and reducing the dynamic error. In pilot studies, a significant reduction in the dynamic error was achieved, and the anemometer’s transfer band was extended by up to forty times. The analysis of problems related to contamination and ice deposition on the rotor in cup anemometers was discussed in article [66]. The research results show that the indications of anemometers in conditions of 50% coverage of the sensor with sediments may be underestimated by 10%. The extensive development of unmanned aerial vehicles means that they are becoming carriers of measuring equipment. A vane anemometer designed to be placed on a flying drone was described in article [67]. The design of the anemometer rotor in this device is inspired by the profile of an owl’s wings, and a triboelectric generator was used to convert the rotor speed into a measurement signal. This generator also supplies the anemometer’s electronic systems. The use of a cup anemometer to measure wind speed in a stratospheric balloon mission is the subject of article [68]. The aim of the research was to analyze the possibilities of using cup anemometers to measure wind speed in relation to a flying object. The wind speed was measured with a balloon rising to a height of 18 km. The research confirmed the possibility of using this type of anemometers in conditions of changing atmospheric density.

The examples presented above are evidence of the continuous and multidirectional improvement of measurement techniques using tachometric anemometers. The authors of this article took up the challenge of designing a tachometric anemometer for measuring wind speed and direction, which does not contain electronic components and systems or power supply systems in the measurement area. Such requirements apply, for example, to anemometers exposed to extremely unfavourable environmental operating conditions, exposed to direct atmospheric discharges, devices meeting strict intrinsically safe requirements, and anemometers for special military applications or operating in the presence of very strong electromagnetic fields. An example is the need to monitor wind in the surroundings of very high antenna radio masts. In the years 1974–1991, there was a long-wave, transmitting radio mast in Konstantynów, Poland, with a height of over 646 m [69]. At that time, it was the tallest engineering structure in the world. During maintenance work, as a result of human negligence, the mast collapsed on 8 August 1991. Equipping such structures with anemometric measurement systems that can operate in the presence of extreme electromagnetic fields may be one of the elements increasing the level of operational safety.

The inspiration for the development of the innovative anemometer presented by us was article [70]. In this article, the authors describe a vane anemometer with an original system for measuring rotor speed and transmitting measurement data. The measuring element of the anemometer is a rotor placed in the flow, the blades of which reflect light. The light incident on the rotor is supplied by an optical fibre, and the reflected signal returns by the same optical fibre to the measuring system placed outside the measuring area. In this solution, the sensor assembly placed in the measuring area does not contain power supply elements or electrical circuits. The measurement signal can be transmitted over long distances.

The novelty of the anemometer described in this article consists of the development of a design intended for measuring both wind velocity and direction, with the impulse measurement signal from the sensor assembly to the measuring station transmitted by optical fibre. The pulse frequency is a function of the flow velocity, and the duty cycle is a function of the wind direction. The sensor assembly does not have electronic systems and power supply systems. The design of the device is simple, durable, and resistant to environmental conditions. The device’s transducer is an original solution, and the developed design, apart from special exploitation conditions, can also be used in extensive multi-point measurements, in which the measurement signals from the sensors are transmitted via optical fibres over long distances to a common measuring station.

## 2. Concept of an Anemometer with Optical-Fibre Transmission of the Measurement Signal

**Figure 1:**
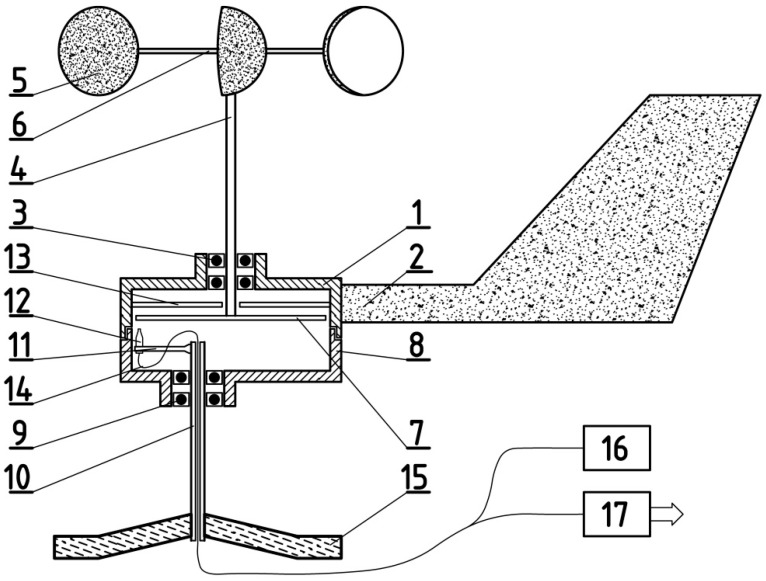
The concept of an anemometer with fibre-optic transmission of the measurement signal: 1—upper housing; 2—rudder; 3—turbine bearing; 4—turbine axis; 5—turbine cups; 6—supports; 7—impulse disc; 8—lower housing; 9—housing bearing; 10—housing axis; 11—bracket; 12—optical-fibre tip; 13—auxiliary disc; 14—optical fibre; 15—base; 16—light source; 17—light pulse detector and electronic system.

The assumptions made in the development of the anemometer concept were that the anemometer should enable the measurement of wind velocity and direction, and that the anemometer sensor assembly placed in the measurement area does not use electronic components or power supply systems. In addition, the construction of the anemometer should be as simple, durable, and resistant to environmental conditions as possible. As a solution to this task, the concept of a tachometric anemometer was selected, in which optical-fibre transmission of the signal from the measurement area to the measurement station located outside this area was used. An original concept was proposed: the measurement signal transmitted by optical fibre from the anemometer sensors to the measurement station would be an impulse signal, the frequency of which would be a function of wind velocity, and the duty cycle would be a function of wind direction. The developed anemometer concept is shown in Figure 1.

The tachometric cup anemometer with a flow direction indicator and fibre-optic transmission of the measurement signal is constructed of a closed, two-part housing in a shape similar to a cylinder. A flow direction rudder 2, made in the shape of a vertical aerodynamic airfoil, is attached to the upper part of the housing 1 from the side. In the upper plane of the housing 1, centrally, coaxially with the housing, a vertical turbine axis 4 is placed in bearing 3. To the axis 4, the cup measuring turbine of the anemometer is attached from above. The turbine is constructed of three cups 5, attached to the axis 4 with horizontal supports 6. Inside the housing, at the lower end of the axis 4, an impulse disc 7 is attached horizontally, reflecting light from the lower side. The impulse disc 7 has a shape similar to a star with eight triangular arms. In the base of the lower part of the housing 8, in bearing 9, a vertical pipe 10 is placed, constituting the rotational axis of the anemometer housing. The rotational axis of the anemometer housing 10 is shifted horizontally relative to the turbine axis 4. A bracket 11 is attached horizontally to the upper end of the axis 10, inside the housing, and a measuring tip of the optical fibre 12 is placed at its end. It is directed upwards, towards the impulse disc 7. Additionally, above the disc 7, inside the upper part of the housing 1, a circular auxiliary disc 13 is attached to the housing from below. This disc is divided into halves due to its optical properties. One half reflects light well from below, and the other half absorbs light. The dividing line of the auxiliary disc 13 into halves coincides with the position of the rudder 2.

The lower end of the rotational axis of the anemometer housing 10 is attached to the base 15, fixed in the measurement area. The wind direction is determined relative to this base. The optical fibre 14 is led out of the lower part of the housing 8 through an opening inside the axis 10, while the other end of the optical fibre 14 is led out of the measurement area to the measurement station. In the measurement station, the optical fibre is connected to the electronic system of the anemometer, containing a light source 16 and an electronic system 17, which is a light pulse detector together with a microprocessor system for processing pulses into the result of the measurement of the velocity and direction of the flow. In the presented anemometer, the rotational speed of the anemometric turbine is measured by illuminating the impulse disc 7 with the optical fibre 14 and receiving the pulses reflected from the arms of the disc. The frequency of these pulses is proportional to the rotational speed of the turbine. In the microprocessor system 17, the frequency of the pulses reflected from the arms of the impulse disc 7 is measured, and the flow velocity is calculated on its basis. Data from the calibration of the anemometer in a wind tunnel in a flow with set, known velocities are used here. The calibration function determines the dependence of the flow velocity on the frequency of the reflected signal.

**Figure 2:**
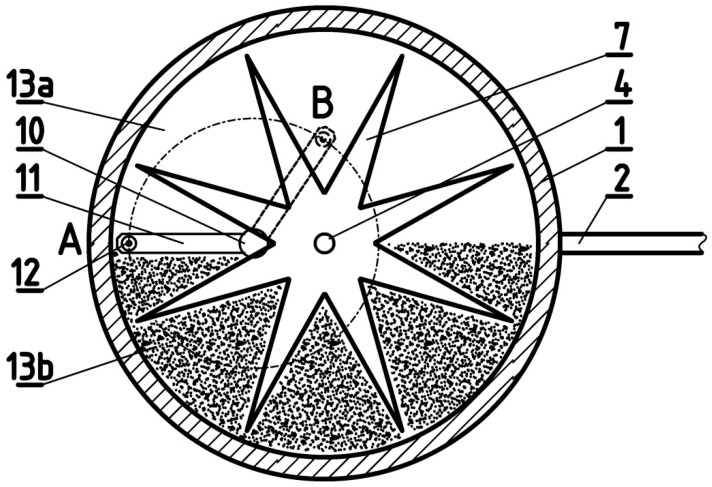
The inside of the anemometer from the side of the impulse disc: 1—upper housing; 2—rudder; 4—turbine axis; 7—impulse disc; 10—housing axis; 11—bracket; 12—optical fibre tip; 13a—half of the auxiliary disc reflecting light; 13b—half of the auxiliary disc absorbing light.

Figure 2 shows the inside view of the anemometer from the side of the impulse disc 7. The purpose of this figure is to explain the concept of flow direction measurement. The horizontal displacement of the anemometer housing axis 10 and the turbine axis 4 relative to each other plays an important role here.

The change in the position of the rudder 2 together with the housing, relative to the stationary base of the anemometer 15, causes a relative shift in the measuring tip of the optical fibre 12 in the radial direction of the impulse disc 7. In Figure 2, this shift illustrates the shift in the measuring tip of the optical fibre 12 from point A to point B, with point B being closer to the centre of the impulse disc 7. The shape of the disc 7, which is similar to a star, with arms widening in the direction of the axis of rotation, causes a change in the duty cycle of the signal of the reflected pulses. The duty cycle of the signal increases as the measuring tip of the optical fibre 12 approaches the centre of the disc 7. Thanks to this, the duty cycle, i.e., the ratio of the duration of the signal reflected from the arms of the disc 7 to the full period of the impulse signal returning to the optical fibre, is a function of the position of the rudder 2. This function is ambiguous, because in two symmetrical positions of the rudder 2, relative to the direction defined by the stationary support 11, the reflected signal has the same duty cycle.

In order to distinguish between these two positions, an additional auxiliary disc 13 is used. This disc is attached to the bottom of the upper part of the housing 1, above the impulse disc 7. One half of the disc 13a reflects light well from below, and the other half 13b absorbs light, with the division line of the auxiliary disc 13 coinciding with the plane of the rudder 2. As a result, the reflected light signal can have one of three levels. The level of the signal reflected from the impulse disc 7 is high; the signal reflected from the auxiliary disc 13a has an intermediate level; and in the case of light absorption with half of the auxiliary disc13b, the signal has a low level. These three levels of light intensity are converted in the photoelectric detector of the microprocessor system 17 into three voltage levels and then converted into a digital signal. In the microprocessor system 17, further processing of the digital signal takes place, i.e., the duty cycle of the reflected signal is measured and the signal levels are detected. On this basis, using the calibration function, the flow direction is determined relative to the stationary base of the anemometer 15. The calibration function is determined in a wind tunnel in a flow with given, known directions. It determines the dependence of the flow direction on the duty cycle and the level of the reflected signal.

The presented anemometer concept enables the measurement of flow velocity and direction, whereby in the measuring area containing the turbine and the rudder, the anemometer does not have electronic components and does not require an electrical power supply system, and the measurement signal of flow velocity and direction is transmitted remotely, using a single signal, using an optical fibre. It is possible to use a single optical fibre with a splitter in the measuring system, separating the incident and reflected beams, or a system of two optical fibres, illuminating and receiving.

## 3. Model of Wind Direction Transducer to Signal Duty Cycle

**Figure 3:**
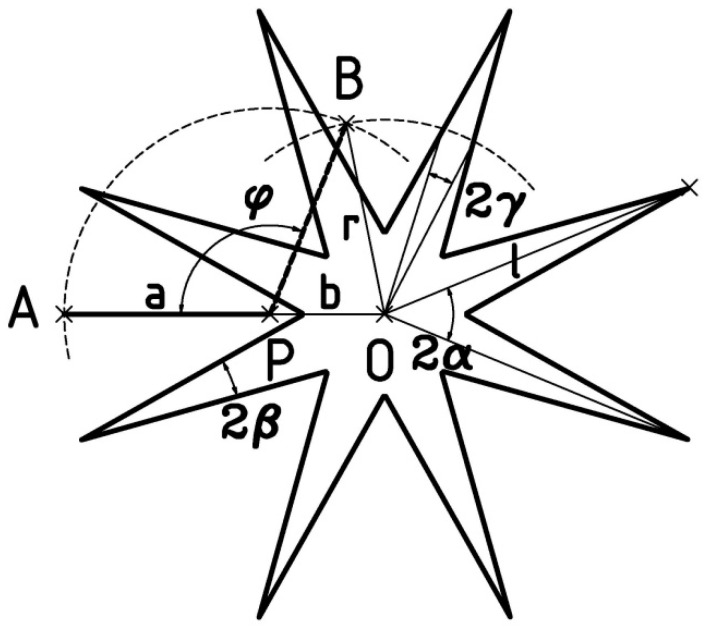
Sketch of the impulse disc and the optical fibre support with important markings.

In the presented anemometer, the wind velocity transducer to the frequency of the impulse signal operates in a conventional manner. The anemometer turbine is placed on a common axis with the impulse disc. The impulse disc is illuminated by an optical fibre, and the signal reflected from the arms of the impulse disc is transmitted by an optical fibre to the light pulse detector. Based on the measurement of the impulse frequency, the flow velocity is determined, and the functional dependence of both quantities is determined in the anemometer calibration process. The innovative solution in the discussed anemometer is the simultaneous use of the wind velocity transducer, also as a wind direction transducer to the impulse signal duty cycle. Figure 3 shows a schematic view of the impulse disc and the optical fibre end support along with the markings of important elements.

In Figure 3, the impulse disc has the shape of a star with triangular arms and rotational symmetry with an angle of 2*α* relative to the axis of rotation at point O. The radius of the disc is l, and the angle at the tip of the disc arm is 2*β*. The optical fibre support of length *a* is attached at point P to the axis of rotation of the anemometer housing. The distance of this axis from the axis of rotation of the impulse disc is *b*. The optical fibre tip is placed at the other end of the support, at point A. The fixed optical fibre support determines the direction of straight line AP, with respect to which the wind direction is measured. Rotation of the anemometer housing by an angle *φ*, related to the effect of the wind on the rudder, causes a relative displacement of the optical fibre tip relative to the centre of the impulse disc from point A to point B. The distance *r* of the optical fibre tip from the centre of the impulse disc is a nonlinear function of angle *φ*. According to Figure 3, the functional relationship can be determined from the geometric analysis:

(1) $$r=\sqrt{a^{2}+b^{2}+2ab\cos\varphi }$$

For a fixed, arbitrary rotational speed of the pulse disc, when the optical fibre tip is located at a distance *r* from the centre of the disc, the duty cycle *η* of the pulse signal reflected from the disc is

(2) $$\eta =\frac{\gamma }{\alpha }$$

where the angle *γ* is half of the angle with the vertex at point O and arms passing through the intersection points of the edge of the impulse disc arm with a circle of radius *r*, as shown in Figure 3. By determining angle *γ* based on trigonometric relations and substituting it into Equation (2), after transformations, we obtain the dependence of the duty cycle *η* on the distance r in the form

(3) $$\eta =\frac{\mathrm{arc sin}\left(l\sin(2\beta )/2r-\sqrt{{\left(l\sin(2\beta )/2r\right)}^{2}-(l^{2}/r^{2}-1){(\sin\beta )}^{2}}\right)}{\alpha }.$$

The system of Equations (1) and (3) gives the dependence of coefficient *η* on angle *φ*, constituting a model of the wind direction transducer to the duty cycle of the signal reflected from the impulse disc for the parameter designations adopted in accordance with Figure 3.

## 4. Construction of the Anemometer Prototype

**Figure 4:**
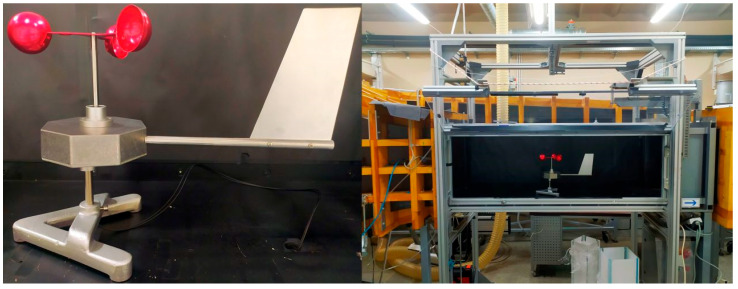
Prototype of anemometer: on the left is the view of the device; on the right is the device placed in the wind tunnel chamber.

In order to verify the concept of a tachometric cup anemometer with a wind direction indicator and fibre-optic signal transmission, a prototype device was built. The view of the prototype is shown in Figure 4.

**Figure 5:**
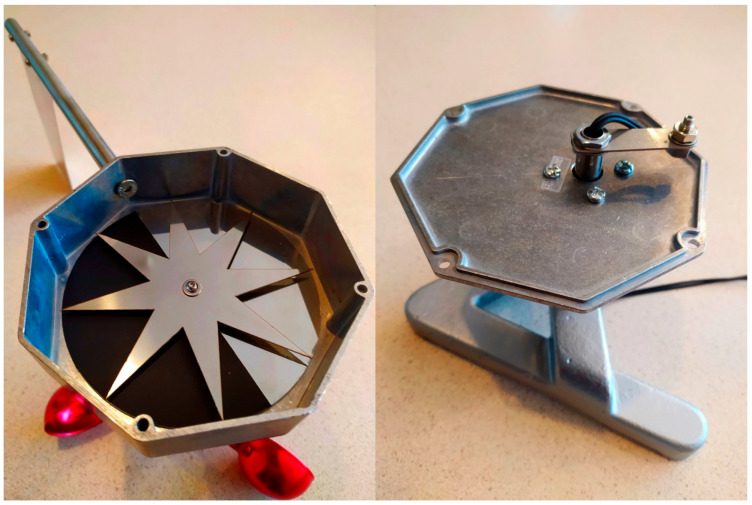
Anemometer measuring transducer: on the left, a view of the upper part of the housing with the impulse disc; on the right, the lower part of the housing with the fibre-optic tip attached to the bracket.

The anemometer measuring transducer was placed in an octagonal, aluminum housing 1590STPC from Hammond, ON, Canada. The rudder was attached to the housing on an aluminum boom. The upper part of the housing contained the turbine rotation axis socket with two ball bearings. The anemometer turbine with three cups was attached to the axis. The lower part of the housing contained the housing rotation axis socket with two ball bearings. The housing rotation axis was attached to the anemometer base. The interior of the anemometer housing with the measuring transducer is shown in Figure 5.

The anemometer impulse disc and the auxiliary disc were made of mirror stainless steel sheet. A black, matte foil was glued onto half of the auxiliary disc. Split polymer optical fibres were used to illuminate the disc and to read the reflected signal. The FRS-610-TZ reflective sensor from Wenzhou DAiGOK Electrical Technology Co., Ltd., Wenzhou, China was used. The light source was an SFH757V optical fibre transmission diode, and the reflected signal detector was an SFH350V optical fibre phototransistor, both from Infineon Technologies, Neubiberg, Germany. The important design parameters of the anemometer prototype are summarized in Table 1.

## 5. Tests of the Anemometer Prototype

In order to verify the correctness of the developed concept of a tachymetric cup anemometer with a wind direction indicator and fibre-optic signal transmission and to assess the obtained parameters, tests of the device were carried out in the SMRI PAS wind tunnel [71,72]. Signals from the detector were observed and recorded using a Rohde Schwartz, Munich, Germany, RM-3004 digital oscilloscope with 10-bit resolution, at a sampling rate of 100 kHz.

**Figure 6:**
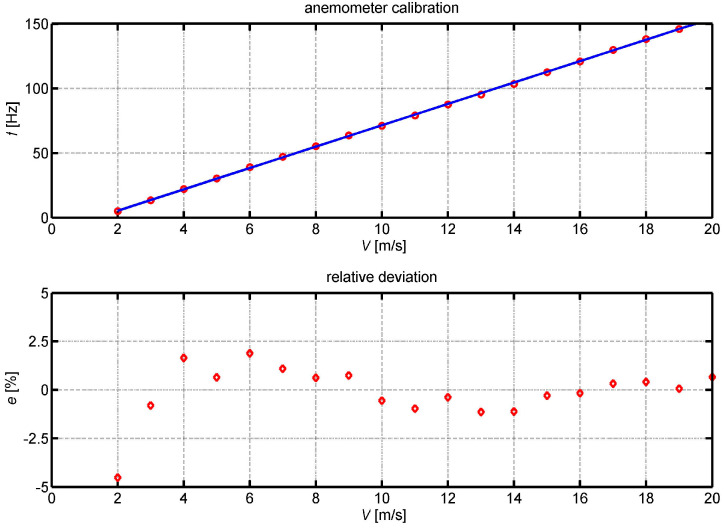
Calibration measurement points of the anemometer prototype with a fitted straight line (upper graph) and the relative deviation in the points from the straight line (lower graph).

The upper graph in Figure 6 shows the measurement points of the prototype calibration in the wind tunnel in the velocity range from 2 to 20 m/s. For the given velocities V, increased by 1 m/s, the averaged frequency of pulses from the impulse disc was recorded. The movement of the anemometer turbine started from a velocity of about 1.2 m/s, and stable measurements were possible from a velocity of 1.8 m/s. A straight line was fitted to the obtained measurement points using least squares method. The parameters of the straight line are the slope *a_r_* = 8.27 Hz/(m/s) and the shift *b_r_* = −11.2 Hz, respectively. The lower graph shows the relative deviation in the measuring points from the straight line. In the low velocity range, the influence of the turbine bearing resistance on the deviation from linearity is visible.

**Figure 7:**
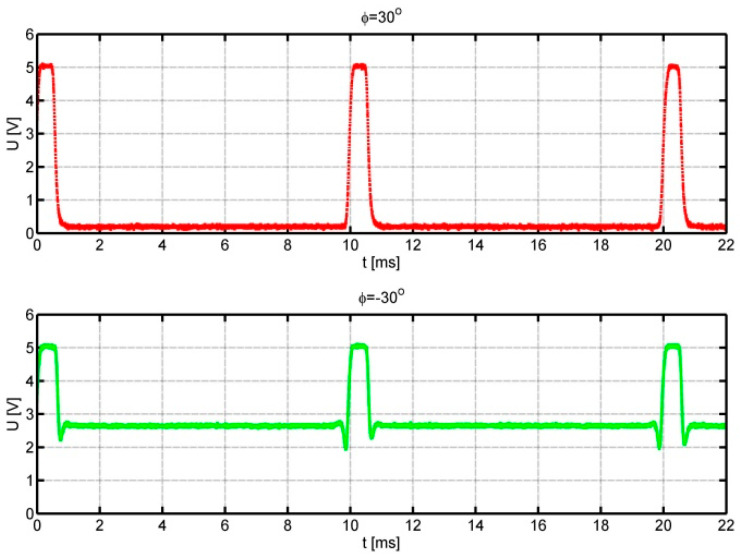
Signals from the anemometer transducer for setting angles *φ* = 30° upper course and *φ* = −30° lower course.

Since wind velocity is measured conventionally, this article focuses on the results of tests of an innovative flow direction transducer. Tests of this transducer were performed by placing the anemometer in the wind tunnel on a rotary table. The table allowed the anemometer base to rotate in the full range of ±180° at set flow speeds and directions. This simulated a change in wind direction. Figure 7 shows the signal waveforms from the anemometer for the setting angles *φ* = 30° upper waveform and *φ* = −30° lower waveform.

**Figure 8:**
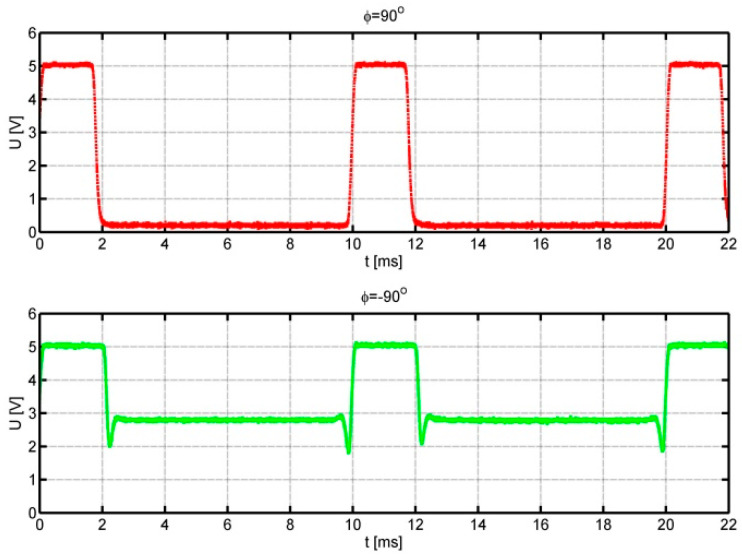
Signals from the anemometer transducer for setting angles *φ* = 90° upper course and *φ* = −90° lower course.

**Figure 9:**
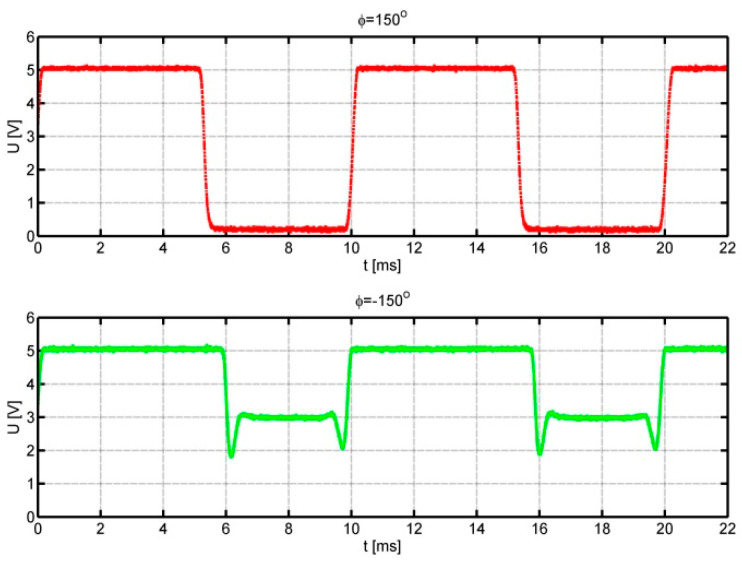
Signals from the anemometer transducer for setting angles *φ* = 150° upper course and *φ* = −150° lower course.

The flow velocity was selected to obtain a pulse frequency of 100 Hz. Positive angles correspond to the movement of the optical fibre tip over the black area of the auxiliary disc. Negative angles, on the other hand, refer to the mirror part of the auxiliary disc. In this case, the increased level of the signal reflected from the auxiliary disc and short spike pulses generated when the optical fibre tip passes over the edge of the impulse disc are visible. The following figures, Figure 8 and Figure 9, show analogous waveforms for setting angles *φ* = ±90° and *φ* = ±150°.

In Figure 7, Figure 8 and Figure 9, we can observe an increase in the value of the signal duty cycle with the increase in the absolute value of the angle between the anemometer base direction in relation to the flow direction. This allows us to determine the function of the dependence of the flow direction on the signal duty cycle. The quality of the obtained signals is very good: in each case, the upper level of the waveform is about 5 V; the intermediate level, 3 V; and the lower level, 0 V. Therefore, the distinction between the flow directions corresponding to positive and negative angles is unambiguous. During the measurements, it was noticed that the value of the signal duty cycle for negative angles is slightly higher than for the analogous angle with a positive value.

**Figure 10:**
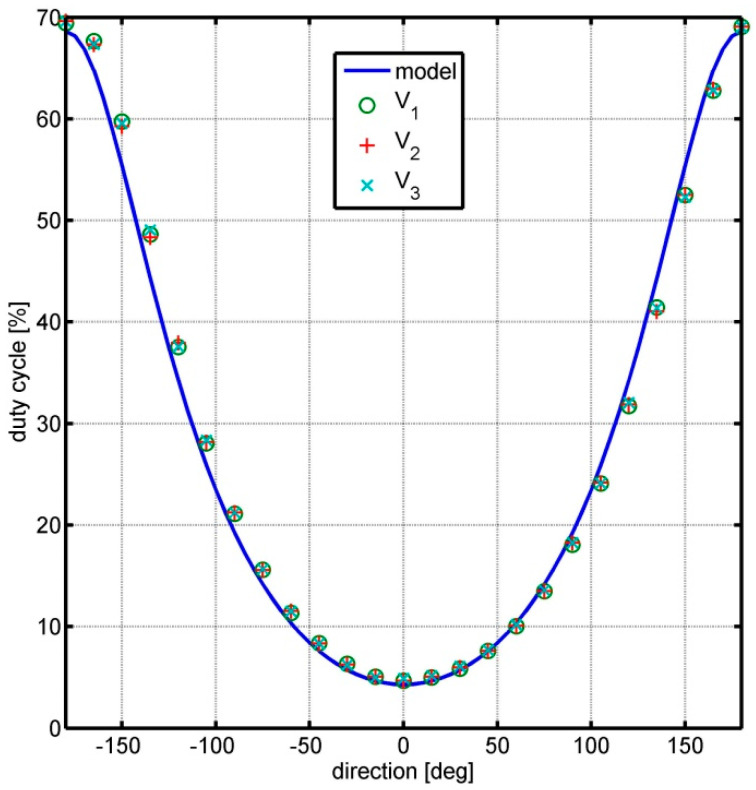
Comparison of the dependence of the signal duty cycle on the flow direction from model (1), (3) with the measurement results for the flow velocity: *V*_1_ = 5 m/s, *V*_2_ = 10 m/s, and *V*_3_ = 20 m/s.

Then, the dependence of the signal duty cycle on the flow direction was measured in the full range of angles. The measurement results were recorded for angle changes every 15°, and the tests were carried out for three different flow velocities *V*_1_ = 5 m/s, *V*_2_ = 10 m/s, and *V*_3_ = 20 m/s. The measurement results are shown in Figure 10. This figure also shows the theoretical course of the dependence of the signal duty cycle on the flow direction, obtained on the basis of model (1), (3), and anemometer parameters according to Table 1.

The measurements carried out show that the duty cycle value for the set angle is practically independent of the flow velocity. In addition, the shape of the experimental characteristic presented in Figure 10 is consistent with the theoretical model. The deviation from the model is a small horizontal shift in the graph of the experimental results, which is most likely caused by an error in assuming a zero angle of the anemometer base position in relation to the flow direction. The obtained characteristic allows for an unambiguous determination of the flow direction based on the measurement of the duty cycle and the signal level. A certain disadvantage of the prototype transducer is the smaller slope of the characteristic near angles *φ* = 0° and *φ* = 180°, which results in lower resolution of the angle measurement in these areas.

## 6. Conclusions

This article presents the concept and construction of the prototype and test results of a tachometric cup anemometer with a wind direction indicator and fibre-optic signal transmission. The main distinguishing feature of the developed anemometer among other devices of this type is the lack of electronic components and power supply systems in the measuring area where the anemometer sensors are placed. This allows the device to be used in special conditions where it is not advisable to use electronic components. The device uses a mechanical design with a dual-function, reflective optical transducer and fibre-optic signal transmission to the measuring station. The transducer is an original solution in which the frequency of the optical output signal pulses is a function of wind velocity, and the duty cycle is a function of wind direction. Initial tests of the prototype confirmed the adopted assumptions. The output signal from the device enables the measurement of wind velocity and direction. In the developed design, wind velocity is measured in a conventional manner and in this respect, further work may concern adapting the shape and dimensions of the turbine to specific metrological requirements. The prototype uses a turbine and turbine bearings that are widely available commercially. The use of these cheap components affects the relatively high turbine start velocity of about 1.2 m/s. To obtain a lower turbine start velocity, it is necessary to select higher quality bearings or increase the turbine size. The shape of the turbine and cups can be further optimized, for example, to reduce the impact of atmospheric precipitation or pollution.

In the prototype solution, the wind direction measurement is performed with a resolution strongly dependent on the wind inflow angle relative to the stationary base. This is illustrated by the nonlinear course of the processing function shown in Figure 10. Therefore, in further work, it is planned to optimize the shape of the impulse disc in order to obtain the most uniform resolution possible in the full range of wind inflow angles. In addition, the use of a single optical fibre with a splitter is considered, which separates the light sent from and received from the sensors in the measuring station. In the initial measurements of the prototype, a shift in the directional characteristic was observed relative to the model. The authors assume that this is related to the low precision of the placement of the auxiliary disc or the end support of the optical fibre. Of course, the cause may be different, e.g., related to the direction of rotation of the measuring disc. This requires further research. However, this does not constitute a problem in the operation of the device, because it is subject to calibration.

Work is also planned to reduce the size of the transducer, which in the prototype device takes up a relatively large volume. To sum up, it seems that it was possible to develop a device that meets the adopted assumptions; the design is simple, reliable, and resistant to environmental conditions, and thanks to this, the potential application area of this innovative solution in special conditions may be significant. It is also possible to use this design in other exploitation conditions in multi-point measurements [73,74,75]. In such a case, it is possible to deploy many simple, cheap, reliable and maintenance-free sensors at selected points of the measurement area and transmit measurement signals exclusively via optical fibres with small losses over long distances. Last but not least, the authors encourage other research teams to develop and present other, smarter concepts of wind speed and direction-measuring devices with optical fibre signal transmission, resistant to environmental conditions, and that do not contain electronic components or power supply systems in the measuring area.

## Figures and Tables

**Table 1:** Design parameters of the anemometer prototype.

Parameter Name	Parameter Value
Anemometer height with base	300 mm
Anemometer length with rudder	400 mm
Anemometer turbine radius	100 mm
Turbine bowl radius	25 mm
Transducer housing radius	70 mm
Transducer housing height	40 mm
Impulse disc radius *l*	60 mm
Disc rotational symmetry angle 2*α*	45°
Disc arm apex angle 2*β*	21°
Distance between rotation axes	15 mm
Optical fibre diameter	1 mm
Transmitting diode supply current	15 mA
Phototransistor supply voltage	5 V
Phototransistor load resistance	10 kΩ

## Data Availability

All data is included in the article.
